# The Genome of *Microthlaspi erraticum* (Brassicaceae) Provides Insights Into the Adaptation to Highly Calcareous Soils

**DOI:** 10.3389/fpls.2020.00943

**Published:** 2020-07-03

**Authors:** Bagdevi Mishra, Sebastian Ploch, Fabian Runge, Angelika Schmuker, Xiaojuan Xia, Deepak K. Gupta, Rahul Sharma, Marco Thines

**Affiliations:** ^1^ Senckenberg Biodiversity and Climate Research Centre (BiK-F), Senckenberg Gesellschaft für Naturforschung, Frankfurt am Main, Germany; ^2^ Goethe University, Department for Biological Sciences, Institute of Ecology, Evolution and Diversity, Frankfurt am Main, Germany; ^3^ Institute of Botany, University of Hohenheim, Stuttgart, Germany

**Keywords:** Brassicales, evolution, genomics, magnesium transporters, *Microthlaspi erraticu*m

## Abstract

*Microthlaspi erraticum* is widely distributed in temperate Eurasia, but restricted to Ca^2+^-rich habitats, predominantly on white Jurassic limestone, which is made up by calcium carbonate, with little other minerals. Thus, naturally occurring *Microthlaspi erraticum* individuals are confronted with a high concentration of Ca^2+^ ions while Mg^2+^ ion concentration is relatively low. As there is a competitive uptake between these two ions, adaptation to the soil condition can be expected. In this study, it was the aim to explore the genomic consequences of this adaptation by sequencing and analysing the genome of *Microthlaspi erraticum*. Its genome size is comparable with other diploid Brassicaceae, while more genes were predicted. Two Mg^2+^ transporters known to be expressed in roots were duplicated and one showed a significant degree of positive selection. It is speculated that this evolved due to the pressure to take up Mg^2+^ ions efficiently in the presence of an overwhelming amount of Ca^2+^ ions. Future studies on plants specialized on similar soils and affinity tests of the transporters are needed to provide unequivocal evidence for this hypothesis. If verified, the transporters found in this study might be useful for breeding Brassicaceae crops for higher yield on Ca^2+^-rich and Mg^2+^ -poor soils.

## Introduction

The plant family Brassicaceae includes many economically important ornamental and crop species. Members of the family are mostly herbaceous, and many can be easily grown in the laboratory, such as *Arabidopsis thaliana*, the first plant to have its genome sequenced, as it is widely used as a model organism for flowering plants. In addition, several other Brassicaceae genomes have been sequenced, facilitating comparative studies (Slotte et al., 2011; Yang et al., 2016; Mandáková et al., 2017). In this study, *Microthlaspi erraticum* of the tribe Coluteocarpeae was targeted for genome sequencing.

Many members of the Coluteocarpeae are able to grow on highly Ca^2+^-rich carbonate soils, and several are heavy metal accumulators, such as *Noccaea caerulescens* (Mandáková et al., 2015). Here, the genome assembly of *M. erraticum* is reported. *M. erraticum* is an interesting plant on which to study environmental adaptation, as it has a wide distribution range throughout warm temperate Europe and Central Asia (Ali et al., 2016a; Ali et al., 2016b; Ali et al., 2017). The species occurs almost exclusively in soil derived from calcium carbonate-rich bedrock und usually grows on well-drained loamy, somewhat open areas (Ali et al., 2017). Similar to *A. thaliana*, *M. erraticum* usually is a winter annual, but has longer seed dormancy, requires vernalisation, and so does not produce a second flowering generation in autumn (Baskin and Baskin, 1979). In nature, the plant hibernates in the rosette stage, but at the southern limits of the distribution, seeds may directly germinate in winter or early spring to produce a flowering plant without going through the rosette stage (unpublished observations). In the laboratory, the time from seed germination to seed maturation is 4–5 months.

Growing on Ca^2+^-rich soil can be challenging for plants, if the soil is at the same time Mg^2+^-deficient, due to the low specificity of channels for bivalent cations. This would lead to an imbalance of Ca^2+^ and Mg^2+^ ions, if the more specific transporters of the *MRS2*/*MGT* family cannot provide enough selectivity to counter this (Schock et al., 2000; Li et al., 2001). There is strong evidence that the *MRS2*/*MGT* family members, and in particular the root-expressed genes, are vital for the fitness of plants in conditions where there is an overwhelming amount of Ca^2+^ in comparison to Mg^2+^ ions (Gebert et al., 2009). As *M. erraticum* is almost completely restricted to such soils derived from very pure Calcium Carbonate rocks, such as the upper Jurassic limestone deposited in the Tethys Ocean (Kimmig et al., 2001), we hypothesized that this could be mirrored in its *MRS2*/*MGT* genes.

*Microthlaspi erraticum* is easy to grow, as it is a rather small plant with a short life cycle, but neither a genome nor molecular tools are available for the species, yet. As the edaphic niche of *M. erraticum* is very well defined, the species provides a good model to investigate environmental adaptation apart from soil effects. Therefore, it was the general aim of this study to provide a well-assembled genome of the species and a specific aim to unravel potential genomic and genetic adaptations to Ca^2+^-rich but Mg^2+^-poor soil, with specific reference to the limiting Mg^2+^ ion uptake.

## Material and Methods

### Plant Material, DNA and RNA Extraction, and Sequencing

For genome sequencing, a six times inbred line of the *Microthlaspi erraticum* was used. This line was named Limburg, after the original collection site beneath the ruins of the stronghold Limburg of Berthold mit dem Barte. He was the founder of the house of Zähringen, an influential medieval house in what are now South-Western Germany, Eastern France, Switzerland, Austria, and Northern Italy. The original mother plant was collected in spring 2007 from beneath the Limburg ruins (near Kirchheim unter Teck, Swabian Alb, Germany) in the flowering stage and kept in a climate chamber at 16°C, 60% humidity and a 14 h light 10 h darkness cycle, taking care that the soil remained always moist. After seed maturation, seeds were collected and air-dried for three months at room temperature. Subsequently, seeds were sown into standard gardening soil and placed into a climate chamber with the same conditions as reported before. Six weeks after germination, when the seedlings had produced a small rosette, plants were transferred to a refrigerator for two weeks. Subsequently, they were brought back to the climate chamber, where they flowered to produce seeds within about two months. From the plants one was separated from the others and used as new mother plant. This way, six generations of selfing were done to create the inbred line Limburg.

For genome sequencing, plants were grown from seeds of the 7^th^ generation as described above, but for two months without vernalisation. Then leaves were collected, surface-sterilized for 1 min in 3% sodium hypochlorite solution with 0.1% Tween, and rinsed in sterile water to remove the disinfectant. Subsequently, DNA and RNA were extracted from this material as described previously (Mishra et al., 2018). As the RNA sequencing was done to guide and improve genepredictions rather than quantifying expression, only a single extraction was done. After checking the integrity and purity of the extracted nucleic acids using agarose gels, DNA and RNA extracts were sent to Eurofins Genomics (Erlangen, Germany) for library preparation (Illumina shotgun libraries with 300 and 800 bp inserts, 3, 8, and 20 kbp LDJ libraries, as well as PacBio shotgun libraries for the RSII instrument) and sequencing.

### Read Trimming and Correction

Genomic paired-end Illumina reads were trimmed for adaptors and bad quality ends using Trimmomatic (v 0.32) (Bolger et al., 2014) with the following parameters: TruSeq3-PE.fa:2:30:10 LEADING:3 TRAILING:3 SLIDINGWINDOW:4:15 MINLEN:60. Afterwards, read pairs containing ambiguous bases were removed from the dataset using a perl script. The remaining reads were evaluated for their quality using FastQFS (Sharma and Thines, 2015) and only paired sequences with an average quality score of 30 and a of length greater than 70 were considered for further analyses. Preliminary contigs were constructed using velvet, version 1.2.10 (Zerbino and Birney, 2008) and subsequently aligned using BLAST against a local NT database (downloaded from NCBI: 3/10/2014). Possible contaminations were found to be *Lachancea thermotolerans*, Cloning vector pUC19 and Synthetic construct clone G1 from *Pseudomonas* species, all probably derived from artefacts during sequencing, as none of these contaminants are present in our laboratory. The reads that were matching to the contamination were removed from the dataset. The cleaned Illumina reads were used to correct the PacBio reads using proovread (Hackl et al., 2014).

Quality control of the transcriptomic single-end Illumina reads were also performed as for the genomic reads but using the “TruSeq3-SE.fa” file for the adapter trimming in trimmomatic.

### Assembly

A hybrid assembly was built on the basis the Illumina-corrected PacBio reads using Canu (Koren et al., 2017). Contigs made up by only 2 to 5 PacBio reads were discarded, but the reads were further used for scaffolding the assembly using SSpace-Long (Boetzer & Pirovano, 2014). SSpace standard (Boetzer et al., 2011) was used afterwards to scaffold the assembly using SG Illumina reads and LJD reads. KGBassebmler (Ma et al., 2012) was used to build a final pseudo-chromosome level assembly, using the karyotype of the related species, *Eutrema salsugineum*, as a template. The genomic data for *M. erraticum* can be found under the following accession numbers – Bioproject ID PRJEB35998, BioSample: SAMEA6449025, SRA: ERS4214584, GenBank assembly: GCA_902728155.2.

### Assembly Assessment, Gene Prediction, and Annotation

The final assembly was subjected to both CEGMA (Parra et al., 2009) and BUSCO (Simão et al., 2015) genome completeness assessments. Transcriptomic reads were mapped onto the assembled genome using tophat2 (Kim et al., 2013). The mapping results were used in the BRAKER1 pipeline (Hoff et al., 2016) which uses GenMark-ET and Augustus to predict the gene space. Homology search for the predicted genes was performed using Blastp against a locally stored NR (non-redundant protein sequences) database (downloaded from NCBI 25/03/2017). Interpro ids of all predicted genes were fetched by running InterPro *via* the webservice option in Blast2GO (Conesa et al., 2005). Inside the Blast2GO framework, blast results and InterPro annotations were merged and GO ids were assigned to the sequences. The most generic GO ids (top level) were removed from the annotation and sequences were further annotated according to their predicted localization. Repeatscout (Price et al., 2005) was used for de-novo identification of repeat elements and for creating a repeat element database. This database was used in repeatmasker (Smit et al., 1996-2010) to predict repeat elements in the genome. Putative repeats were further filtered on the basis of their copy numbers and only those repeats present with more than 10 copies in the genome were annotated as repeats. This way, repeat domain families were identified in *M. erraticum* and four other Brassicaceae genomes (**Table S1**) downloaded from the JGI genome portal (https://phytozome.jgi.doe.gov/pz/portal.html). Interproscan was run over the gene-set from these species and the number of sequences from each species matching to specific repeat domain families were obtained.

### Positive Selection Analysis

The protein and the nucleotide sequences of the one-to-one orthologs were fetched from five Brassicaceae species (*A. thaliana*, *A. lyrata*, *Capsella rubella*, *Eutrema salsugineum*, and *M. erraticum*) for a list of 1:1 orthologs generated using OrthoMCL, v2.0.9 (Li et al., 2003). The protein sequences were aligned using mafft, v7 (Katoh and Standley, 2013) with default parameters. The protein alignment and the nucleotide sequences were used in the program transalign from EMBOSS (version: 6.4.0.0) (Rice et al., 2000) to produce codon alignments. For phylogenetic analyses raxmlHPC-PTHREADS-SSE3 of RAxML v8.1.17 (Stamatakis, 2014) was used with the algorithm parameter -f a, which runs rapid Bootstrap analysis and the search for the best-scoring ML tree in one program run. The substitution model was selected as -m GTRGAMMAI which uses GTR, plus an optimization of substitution rates, plus a GAMMA model of rate heterogeneity, plus an estimation of the proportion of invariable sites. The program was run with 1,000 bootstrap replicates (Felsenstein, 1985). Codon alignments of the coding sequences and newick-formatted phylogenetic trees were used to run positive selection analyses with the codeML module of PAML, v4.8 (Yang, 2007). The site model was run to identify positively selected genes, and the branch-site model was run to identify species-specific positive selection of the genes. As multiple hypotheses were tested in the branch-site model of codeML, *q* values were calculated for false discovery rate (FDR) testing using *q* values (Bass et al., 2015) calculated with Bioconductor in an R environment, v3.4.1.

The same approach as followed for the one-to-one orthologs in the five species, was also used for their *MRS2*/*MGT* genes to analyse the selection pressure on the Mg^2+^ transporters.

### Annotations of *MRS2*/*MGT* Mg^2+^ Transporter Genes

Functional *MRS2*/*MGT* Mg^2+^ transport genes from *A. thaliana* were used for the identification of the potential Mg^2+^ transporters in *M. erraticum* by homology search. The criteria for the homology search were as follows: evalue < 10e-5; percentage identity > 50%; length of match > 50%. A phylogenetic tree was constructed using *A. thaliana* Mg^2+^ transporters and their *M. erraticum* orthologs by using mafft v7 (Katoh and Standley, 2013) for multiple alignment and raxmlHPC-PTHREADS-SSE3 of RAxML v8.1.17 (Stamatakis, 2014) with 1,000 bootstraps for the tree construction. All genes were inspected for *MRS2*/*MGT* specific domains and re-annotated according to the results. The *MRS2*/*MGT* genes from *M. erraticum* were blasted against genes from the three additional Brassicaceae genomes considered in this study, i.e. *Arabidopsis lyrata*, *Eutrema salsugineum*, and *Capsella rubella*, and blast hits with an e-value lower than 10e-5 and an at least 50% length match with more than 50% identity were taken as putative *MRS2*/*MGT* genes. Further, the presence of two transmembrane domains was checked using the TMpred at https://embnet.vital-it.ch/software/TMPRED_form.html. The presence of a GMN domain at the end of the first transmembrane domain was checked manually. The *MRS2*/*MGT* genes from the five Brassicaceae genomes were further aligned using mafft v7 (Katoh and Standley, 2013) and a phylogenetic tree was built using the method reported above.

## Results

### Assembly

The 2C value and genome size for *M. erraticum* Limburg had been estimated using flow cytometry with *Glycine max* as size standard (Ali et al., 2016b). The 2C value was 0.44 pg, corresponding to a diploid genome size of 422 Mbp, implying a haploid genome size of 211 Mbp. The hybrid assembly generated using the Illumina and PacBio data was of 170 Mbp in length, out of which 156 Mbp were assembled into 7 pseudo-chromosomes. The rest of the contigs were built into two pseudo-molecules, one with predominantly genic regions and the other with predominantly non-genic regions. The placement of 1,940 scaffolds within the 9 units and the sizes of the pseudo-chromosomes are listed in **Table S2**. Only one gene was not identified when using CEGMA, implying more than 99% completeness of the genic space. The assembled genome had 0.61% of N and the GC content was 37.87%. In the BUSCO analysis out of a total of 1,375 genes that were considered to check the completeness of the gene space, and 98.5% of the genes were present in the genome, out of which 8.2% were duplicated. A total of 0.4% of the genes were present as fragments and a total of 1.1% of the genes were missing. The shotgun Illumina reads were mapped onto the final genome using Bowtie2 (Langmead and Salzberg, 2012) and the reads mapped to 169 Mb of the assembled genome. The PacBio reads were also mapped back to the genome using Blasr (Chaisson and Tesler, 2012) and the reads mapped back to 160 Mb of the assembled genome. Out of 170 Mb, 159 Mb of the total genome were mapped by both Illumina short reads and PacBio reads.

### Genomic Features

The number of genes predicted in *M. erraticum* was 51,309 (with coding space of 55.19Mb), which is higher than in other Brassicaceae, but the proportion of coding space to non-coding space in the genome is similar to that of *A. thaliana* (**Table 1**). The 51,309 genes included 1,372 splice variants, resulting in 49,937 unique genic locations out of which 49,060 were complete genes with both start and stop codons and without in-frame stop codons. A total of 34,370 genes were found to have transcript support when transcriptomic reads were mapped to the genes. In the blast2go annotation process, 1,521 genes were not annotated. The number of genes annotated with GO terms of molecular functions, cellular components and biological processes is given in **Figure S1**, with top functions under each category. A total of 35,042 genes was provided with a GO term.

**Table 1:** Details of genome features in respect to size, genes, coding regions, and repeat regions for *M. erraticum* and four other Brassicaceae species.

Genome	Genome size (Mb)	Gene numbers	Coding space (Mb)	Coding space (%)	Simple repeats (%)	Interspersed repeats (%)	Reference
***M. erraticum***	170.42	51309	55.19	32.38	1.3	33.93	This study.
***A. lyrata***	206.66	33132	38.61	18.68	1.51	35.86	Lamesch et al. (2012)
***A. thaliana***	119.66	35386	43.55	36.39	1.59	15.9	Rawat et al. (2015)
***C. rubella***	134.83	28447	35.66	26.45	2.12	16.88	Slotte et al. (2013)
***E. salsugineum***	243.11	29284	36.12	14.86	1.22	51.81	Yang et al. (2013)

The genome of *M. erraticum* contains 34% of Interspersed repeats, a percentage comparable to *A. lyrata* which has 36% of interspersed repeats in its genome. *A. thaliana* and *C. rubella* have a substantially lower percentage of interspersed repeats with 16 and 17%, respectively. The outcrosser *E. salsugineum* has the highest percentage of interspersed repeats with 52% (**Table 1**). All five genomes have a similar percentage of simple repeats with around 2% (**Table 1**) of the genome. Thus, the proportion of the coding space to the genome size in *M. erraticum* is similar to that of selfing plants but the interspersed repeat regions are higher in proportion.

Repeat domain family associated genes known to have role in biotic and abiotic stress (see discussion) were analyzed in the five species used for comparisons. *M. erraticum* has substantially more members of Pentatricopeptide (PPR), Leucine-rich repeat (LRR and LRR-2) and Kelch repeat domain families in comparison to all other species in this study while having similar number of genes in the Armadillo, HEAT, Ankyrin, Tetratricopeptide (TPR), RCC1, WD40 repeat domain families (**Figure S2**).

Of the 819 LRR and LRR-2 genes of *M. erraticum*, the majority are F-box proteins (342 proteins) and receptors (314 proteins). Out of the latter, 110 are classified as probable serine threonine-kinase receptors and several as involved in plant defence, acting as disease resistance genes (61 proteins), out of which 25 are annotated as nucleotide-binding site (NBS)-leucine-rich repeat (LRR) domain containing R genes. Apart from the functional annotation of Blast2Go, a separate domain search revealed that a total of 49 genes have both NBS and LRR domains. A similar search in *A. thaliana* indicated the presence of 40 NBS and LRR domain containing genes. The detailed numbers of genes containing NBS and LRR domains in five species are presented in the **Table 2**.

**Table 2:** Numbers of genes in five Brassicaceae genomes that contain nuclear binding site (NBS) and/or leucine-rich repeat (LRR) domains.

Genome	NBS domain	LRR domains	NBS+LRR domain
***M. erraticum***	279	819	49
***A. lyrata***	126	417	19
***A. thaliana***	208	558	40
***C. rubella***	139	590	30
***E. salsugineum***	129	522	30

In *M. erraticum*, out of 259 proteins containing the Kelch repeat domain, 206 are F-box proteins (FBK). The majority of the non-F-box Kelch repeat domain proteins belonged to Galactose oxidase Kelch repeat superfamily and few are receptors to different chemicals and viral substrates. FBK proteins in *M. erraticum* are around twice in number when compared to the other species in this study.

### Positive Selection Analyses of the One-to-One Orthologs

In the test of positive selection using the site model from codeML, out of 6,725 one-to-one core orthologs, 92 were inferred as positively selected, with at least one amino acid being positively selected according to Bayes Empirical Bayes (BEB) analysis (Yang et al., 2005) with *p* > 95%. An additional 305 genes had omega values > 1, but no amino acid position in those genes had a significant BEB value. In the test of positive selection using the branch-site model, positively selected genes in individual species were identified. **Figure S3** shows a bar plot showing the numbers of positively selected genes in the individual species. Though the number of positively selected genes in *M. erraticum* is slightly higher than the other species, the difference is not pronounced.

### *MRS2*/*MGT* Gene Family (Mg^2+^ Ion Transporters)

**Figure 1:**
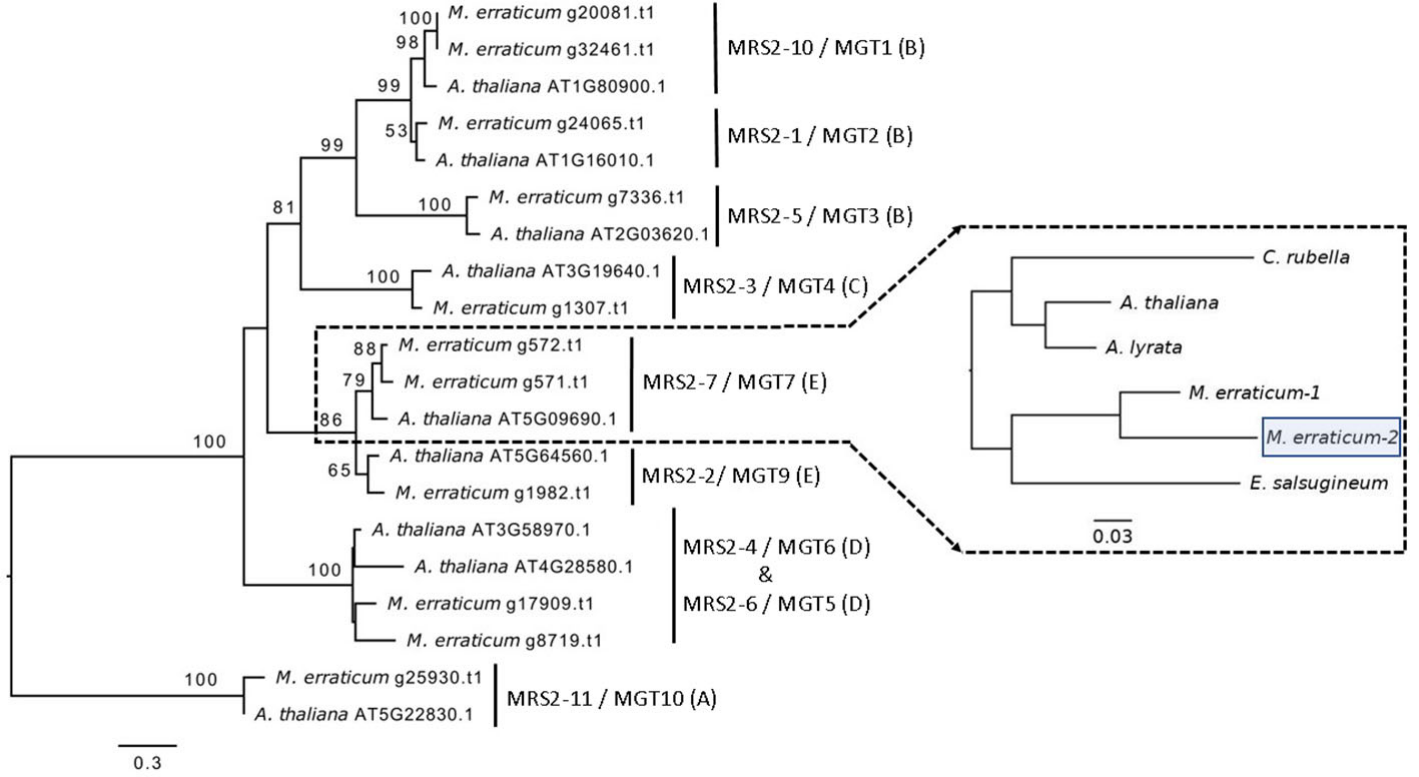
Phylogenetic tree of 20 *MRS2*/*MGT* genes (11 from *M. erraticum* and 9 from *A. thaliana*) with the main monophyletic groups highlighted as A–E, showing duplication of *MRS2-7*/*MGT7* and *MRS2-10*/*MGT1* genes in *M. erraticum.* An inset of the *MRS2-7*/*MGT7* clade with five Brassicaceae species is shown, which was used for a positive selection analysis. One of the two *MRS2-7*/*MGT7* genes in *M. erraticum* was positively selected and is highlighted in the figure.

In the Blast2GO pipeline, 13 genes were assigned to the *MRS2*/*MGT* gene family out of which two were discarded, one being an isoform giving rise to the same gene product and the other lacking a functional GMN domain, resulting in a total of 11 *MRS2*/*MGT* genes in *M. erraticum* (**Table S3**). All of these 11 genes had two transmembrane domains and one GMN domain; and all were homologous to the *MRS2*/*MGT* genes in *A. thaliana*. The genes in *MRS2*/*MGT* gene family in plants are grouped into 5 clades, named from A to E. In *M. erraticum* Clade-A and Clade-C have 1 gene each and Clade-B, Clade-D, and Clade-C have 4, 2 and 3 genes in each, respectively. Details on these genes are presented in **Table 3**. The *MRS2*/*MGT* genes were also mined from the other Brassicaceae genomes used in this study and details of these genes are given in **Table S2**. *Microthlaspi erraticum* had the highest number of *MRS2*/*MGT* genes in comparison to the other Brassicaceae species. A phylogenetic tree using all the mined *MRS2*/*MGT* genes from *M. erraticum* and related species is presented in the **Figure S4**. Interestingly, duplications in two clusters of *MRS2*/*MGT* genes were observed for *M. erraticum* and one of these genes was positively selected (**Figure 1**). 

**Table 3:** Details of the *MRS2*/*MGT* genes identified in *Microthlaspi erraticum* genome.

Gene ID	Annotation	peptide length	# exons	# trans-membrane domains	GMN domain	clades	location on chromosomes
**g25930.t1**	MRS2-11/*MGT*10	456	13	2	Yes	clade-A	ChrUd1
**g20081.t1**	MRS2-10/*MGT*1	443	3	2	Yes	clade-B	ChrUd1
**g32461.t1**	MRS2-10/*MGT*1	411	4	2	Yes	clade-B	5
**g7336.t1**	MRS2-5/*MGT*3	399	6	2	Yes	clade-B	ChrUd1
**g24065.t1**	MRS2-1/*MGT*2	443	4	2	Yes	clade-B	1
**g1307.t1**	MRS2-3/*MGT*4	471	6	2	Yes	clade-C	ChrUd1
**g8719.t1**	MRS2-4/*MGT*6	414	3	2	Yes	clade-D	ChrUd1
**g17909.t1**	MRS2-4/*MGT*6	425	3	2	Yes	clade-D	5
**g571.t1**	MRS2-7/*MGT*7	616	14	2	Yes	clade-E	6
**g572.t1**	MRS2-7/*MGT*7	384	11	2	Yes	clade-E	6
**g1982.t1**	MRS2-2/*MGT*9	396	10	2	Yes	clade-E	2

## Discussion

### Genome Size and Gene Space

The assembled genome size of *Microthlaspi erraticum* is 170.42 Mb, which is larger than the genomes of other selfing plants included in this study, *A. thaliana* (119.66 Mb) and *Capsella rubella* (134.83 Mb), but is smaller than the genomes of outcrossers, *Arabidopsis lyrata* (206.66 Mb) and *Eutrema salsugineum* (243.11 Mb). Generally, selfing plants have less transposable elements in comparison to outcrossing plants, causing genome size differences between them (Johnston et al., 2005; Wright et al., 2008). There is evidences that many repeat domain proteins have roles in coping with abiotic stress conditions such as the Armadillo gene family in rice (Sharma et al., 2014), the mitochondrial PPR-PGN protein (PPR repeat protein for germination on NaCl) in *A. thaliana* (Laluk et al., 2011), and proteins of the LRR repeat family in *A. thaliana* (Osakabe et al., 2005; Park et al., 2014). The presence of excess of interspersed repeats in the genome of *M. erraticum* might indicate possible genomic and genic rearrangements in *M. erraticum* that might have emerged to cope with the stress resulting from the harsh abiotic factors the plant is experiencing in its habitat. In line with this assumption, compared to other species, the proportion of genic repeats in *M. erraticum* was found to be substantially higher (**Figure S5**).

### Genes

Positive selection analyses of one-to-one orthologous genes does not suggest any drastic difference in level of positive selection in *M. erraticum* in comparison to the other species in this study. A comparison of the number of members in the 10 repeat domain family genes that have a known role in biotic and abiotic stress conditions, indicates similar number of genes for the six species in all families except PPR, LRR, LRR-2, and Kelch, for which *M. erraticum* has a substantially higher number of genes.

In *M. erraticum,* 672 genes are found to have PPR repeats. Proteins from the PPR repeat family have a role in growth and development of plants, but many PPR proteins are also known to be biotic and abiotic stress regulators. They have roles in high salinity, drought, and cold stress tolerance (Laluk et al., 2011; Yuan and Liu, 2012; Zhu et al., 2014; Jiang et al., 2015). As *M. erraticum* grows in environments that face both frost in winter and drought during seed maturation, it could be possible that this is reflected by the high PRR gene content.

In *M. erraticum,* 819 genes are classified to belong to the Leucine-rich repeat family proteins (LRR and LRR-2), which is far more than in the other species analysed (**Figure S5**). LRR and LRR-2 family genes are signalling molecules in plants and also have a role in plant development (Hsu et al., 2000) and pathogen defence (Li and Chory, 1997; Deyoung and Clark, 2008). Expression level studies of LRR repeat domain proteins in rice (Park et al., 2014) and *Arabidopsis* (Osakabe et al., 2010) have shown that LRR repeat proteins also positively regulate genes involved in coping with various abiotic stress conditions. This is further supported by Van der Does et al. (2017), who found that MIK2/LRR-KISS is involved in sensing cell-wall integrity changes in response to both biotic and abiotic stress in line with LRR-receptors acting to recognise both pathogen associated molecular patterns and danger signals (Boller and Felix, 2009). It is tempting to speculate that the very rich LRR complement of *M. erraticum* is not only due to the frequent presence of downy mildew in its populations, but also due to the often open slopes on which *M. erraticum* occurs with frequent soil movements, which might need an enhanced and precise danger recognition that responds to root injury. However, more detailed analyses and functional tests will be needed to provide a solid ground to investigate this interesting pattern further.

Kelch repeat domains are found mostly in the C-terminus of F-box proteins. F-Box coupled Kelch (FBK) proteins are abundant in plants, with very few non-plant representatives (Schumann et al., 2011), and are associated with several vital plant molecular mechanisms. Many are associated with growth and development (Zhang et al., 2013), secondary metabolism, Circadian clock and photoperiodic flowering (Nelson et al., 2000) by taking part in signal transduction in various pathways. They also play a role in coping with abiotic stress conditions (Jia et al., 2012; Chen et al., 2014). The finding that *M. erraticum* contains about twice as many FBK genes (206) as the other plants investigated in this study might again indicate an adaptation to stressful environmental conditions. This is also reflected by the fact that *M. erraticum* is often among the few or even the only plant that is present in some open slopes it colonises (unpublished observations).

### Uptake and Transport of Cations in *M. erraticum* in Calcium-Rich Soil

#### Uptake and Transport of Ca^2+^ Ions

Two‐pore channel 1 (TPC1) is responsible for transport of Ca^2+^ from vacuoles to the cytoplasm and expression of TPC1 regulates the storage capacity of Ca^2+^ in the vacuoles (Pottosin et al., 2009; Gilliham et al., 2011). Each of the species that we included in this study have one gene each that codes for TPC1. The more specific Cyclic Nucleotide-Gated Ion Channel, AtCNGC2 has been reported to have crucial role in adaptation to Ca^2+^ Stress in plants (Chan et al., 2003 & Wang et al., 2016). AtCNGC2 is coded by a single gene in *A. thaliana* and has one homolog in each *M. erraticum* and other Brassicaceae species included in this study. Also for other Ca2+ channels, no unusual variation was found. This probably reflects the high Ca^2+^ supply that has also been described to be advantageous (Yamazaki et al., 2000; Sugimoto et al., 2010) and thus does not necessitate enhanced channel specificity, duplication or other forms of adaptation.

#### Uptake and Transport of Mg ^2+^ ions

As *Microthlaspi erraticum* is found almost exclusively in soil derived from Ca^2+^-rich but Mg^2+^ -poor bedrock (Kimmig et al., 2001; Ali et al., 2017), we speculated that an adaptation regarding the targeted uptake of Mg^2+^ might have evolved that gives the species an evolutionary advantage over other Brassicaceae species. Mg^2+^ is an essential bivalent ion with vital functions as a co-factor with ATP in various enzymatic reactions and as central ion in the porphyrine ring of chlorophyll molecules.

Different types of Mg^2+^ transporters interactively transport the ion across membranes in plant tissues to maintain homeostasis. In the presence of excessive Ca^2+^ ions in soil solution, specialized Mg^2+^ transporters might be playing a major adaptive role. The *MRS2*/*MGT* (Schock et al., 2000; Li et al., 2001) gene family is known to harbour various proteins that transport Mg^2+^ across membranes. *MRS2*/*MGT* Mg^2+^ transporters have two trans-membrane domains at the C-terminus with a characteristic GMN domain at the end of the first trans-membrane domain. In *M. erraticum* 11 potential *MRS2*/*MGT* genes were identified with two transmembrane domains and a GMN motif. In comparison *A. thaliana* and rice code for 10 such genes, while 9 are reported from maize (Li et al., 2016). The *MRS2*/*MGT* gene *AtMRS2-10*, has been shown to be expressed in the root in the plasma membrane (Gebert et al., 2009). For this gene, two homologs are found in *M. erraticum*, meaning that this gene has been duplicated. All other species have only one homolog except *C. rubella* in which no homolog for *MRS2-10*/*MGT1* was found (**Figure S4**). Single knock-out experiments and a double knock-out of *MRS2-1*/*MGT2* and *MRS2-5*/*MGT3*, as well as *MRS2-5*/*MGT3* and *MRS2-10*/*MGT1* (Gebert et al., 2009), had no visible effect under normal growth conditions, pointing at functional redundancy of the *MRS2* gene family members. In a phylogenetic analysis, it was shown that *MRS2-1*/*MGT2* and *MRS2-10*/*MGT1* form a sub-clade of the family and plants with double knock-out of *MRS2-1*/*MGT2* and *MRS2-10*/*MGT1* have a high demand of Mg^2+^ for normal growth (Lenz et al., 2013). Thus, the presence of a third member in this sub-clade might indicate a genomic adaptation to the high Ca^2+^/low Mg^2+^ soil condition.

*MRS2-4*/*MGT6* and *MRS2-6*/*MGT5* form a subclade in *A. thaliana*. All species investigated in this study have two genes in this subclade except *E. salsugineum* which has only one. Considering the phylogenetic distance of this gene from the two *A. thaliana* members of this group, it can be assumed that *E. salsugineum* is missing *MRS2-6*/*MGT5*. *MRS2-4*/*MGT6* had previously been identified to localize on either chloroplast or mitochondria in shoots (Gebert et al., 2009; Conn et al., 2011), but a later study has identified this gene to be localised in root plasma membrane under lowered Mg^2+^ conditions and that in Mg^2+^-deficient experimental conditions the transcript levels of this gene in the root increased eight-fold (Mao et al., 2014). Thus, it seems possible that the retaining of the duplication of *MRS2-4*/*MGT6* in *M. erraticum* is adventageous in Mg^2+^-poor conditions.

Another subclade in *A. thaliana* comprise of *MRS2-2*/*MGT9*, *MRS2-7*/*MGT7* and *MRS2-8*/*MGT8*. In some ecotypes in *A. thaliana MRS2-8*/*MGT8* has been found to be a pseudogene (Gebert et al., 2009). *MRS2-7*/*MGT7* from this clade, an ER-localized transporter, is known to be expressed in roots and to promote growth in plants growing in Mg^2+^ deficient soil (Gebert et al., 2009). Its expression was found to be essential for germination in a solution culture system and for normal growth in low Mg^2+^ conditions (Gebert et al., 2009; Conn et al., 2011). In our analyses, we found a duplication of *MRS2-7*/*MGT7* in *M. erraticum* in this clade (**Figure 1**). One of these genes in *M. erraticum* was found to be positively selected with significant *p*- and *q*-values in the branch site model of codeml (**Figure 1**). As *MRS2-7*/*MGT7* has shown to be important in Mg^2+^-deficient conditions, we speculate that its duplication might again be an adaptation of *M. erraticum* to Ca^2+^-rich but Mg^2+^ -poor soils.

## Conclusion

In conclusion, the genome sequence of *M. erraticum* provided several indications of adaptation to stressful abiotic conditions, which is in line with its ephemeral growth in habitats with shallow soil and little vegetation cover, exposing it to a variety of adverse environmental conditions. Probably the most striking characteristic of the preferred habitat of *M. erraticum* is that its soil is derived usually from white Upper Jurassic limestone, a bedrock that is extremely rich in Ca^2+^ but rather poor in Mg^2+^ (Kimmig et al., 2001), creating an environment in which vital Mg^2+^ ion uptake is difficult to achieve. The duplication of two Mg^2+^ transporters that have been shown to be important for Mg^2+^ uptake in Mg^2+^-deficient conditions is indicate an adaptive response to this. Further experiments are necessary to carry out transgenic and affinity assays to underpin this assumption. Should heterologous expression and affinity experiments support this hypothesis, the *MRS2*/*MGT* family of *M. erraticum* could be an interesting target for improving crop yield on highly calcareous soils.

## Data Availability Statement

The datasets generated for this study can be found under the accession number NCBI PRJEB35998 (https://www.ncbi.nlm.nih.gov/bioproject/PRJEB35998).

## Author Contributions

MT conceived the study. MT, AS, and FR created the Limburg inbred line. SP and XX performed laboratory experiments. BM, RS, and DG analyzed the genome. BM and MT interpreted the data and wrote the manuscript, with contributions from the other authors.

## Funding

This study has been supported by LOEWE in the framework of the Centre of Translational Biodiversity Genomics and by the Max Planck Society through a fellowship awarded to MT.

## Conflict of Interest

The authors declare that the research was conducted in the absence of any commercial or financial relationships that could be construed as a potential conflict of interest.
