# Supplementary material for: The Genome of Microthlaspi erraticum (Brassicaceae) Provides Insights Into the Adaptation to Highly Calcareous Soils
Source: Front Plant Sci. 2020 Jul 3;11:943. doi: 10.3389/fpls.2020.00943 (PMC7350527; doi:10.3389/fpls.2020.00943)
Supplement: Supplementary file 1 [file Table_1.docx]

**Table S1** Details and sources of the five genomes used in this study..

| Genome | Assembly versions | References |
| --- | --- | --- |
| *M. erraticum* | V1.0 | This study. |
| *A. thaliana* | TAIR10 (JGI portal) | Lamesch *et al*. (2012) |
| *A. lyrata* | V1 (JGI portal) | Rawat *et al.* (2015) |
| *C. rubella* | V1.0 (JGI portal) | Slotte *et al.* (2013) |
| *E. salsugineum* | V1.0 (JGI portal) | Yang *et al.* (2013) |

**Table S2** Total size of the pseudo-chromosomes and numbers of scaffolds assigned to them.

| Chromosomes | # Scaffolds | Size (in Mbp) |
| --- | --- | --- |
| Chr1 | 77 | 16.87 |
| Chr2 | 64 | 16.15 |
| Chr3 | 65 | 13.36 |
| Chr4 | 47 | 10.53 |
| Chr5 | 47 | 12.33 |
| Chr6 | 60 | 15.99 |
| Chr7 | 73 | 17.48 |
| ChrUdGenic | 545 | 53.08 |
| ChrUdNonGenic | 962 | 14.58 |

**Table S3** Details of the *MRS2*/*MGT* gene homologs in five Brassicaceae genomes.

|  | Blast (<10e-5 e value, >50% identity & length coverage) | Presence of 2 transmembrane domain & GNM motif |
| --- | --- | --- |
| *A. thaliana* | 10 | 9 |
| *A. lyrata* | 9 | 9 |
| *C. rubella* | 8 | 8 |
| *E. salsugeneum* | 10 | 10 |
| *M. erraticum* | 13 | 11 |
