# Supplementary figures and images for: The Genome of Microthlaspi erraticum (Brassicaceae) Provides Insights Into the Adaptation to Highly Calcareous Soils
Source: Front Plant Sci. 2020 Jul 3;11:943. doi: 10.3389/fpls.2020.00943 (PMC7350527; doi:10.3389/fpls.2020.00943)

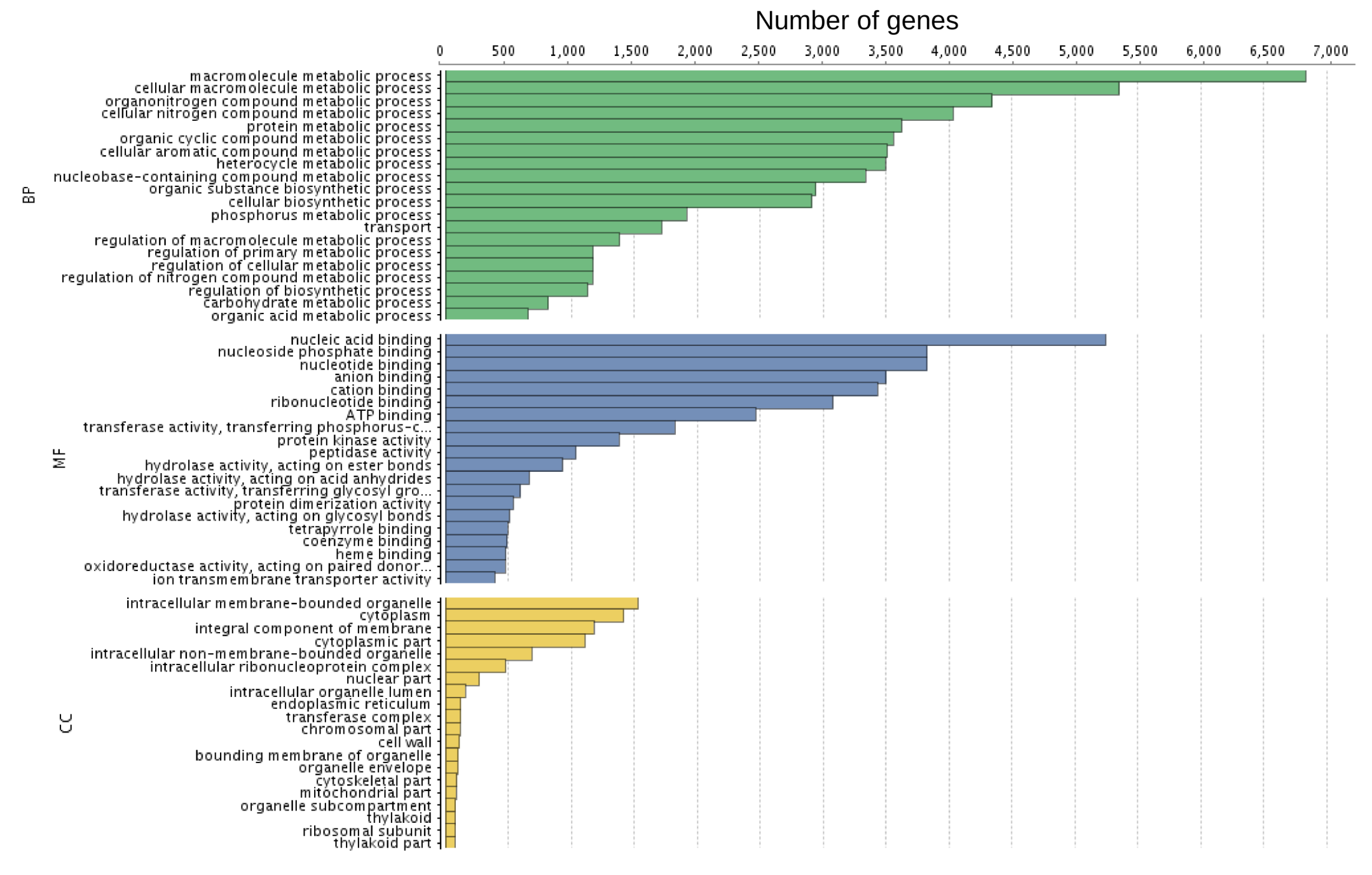

Supplement: Supplementary file 2 [file Image_1.tiff]

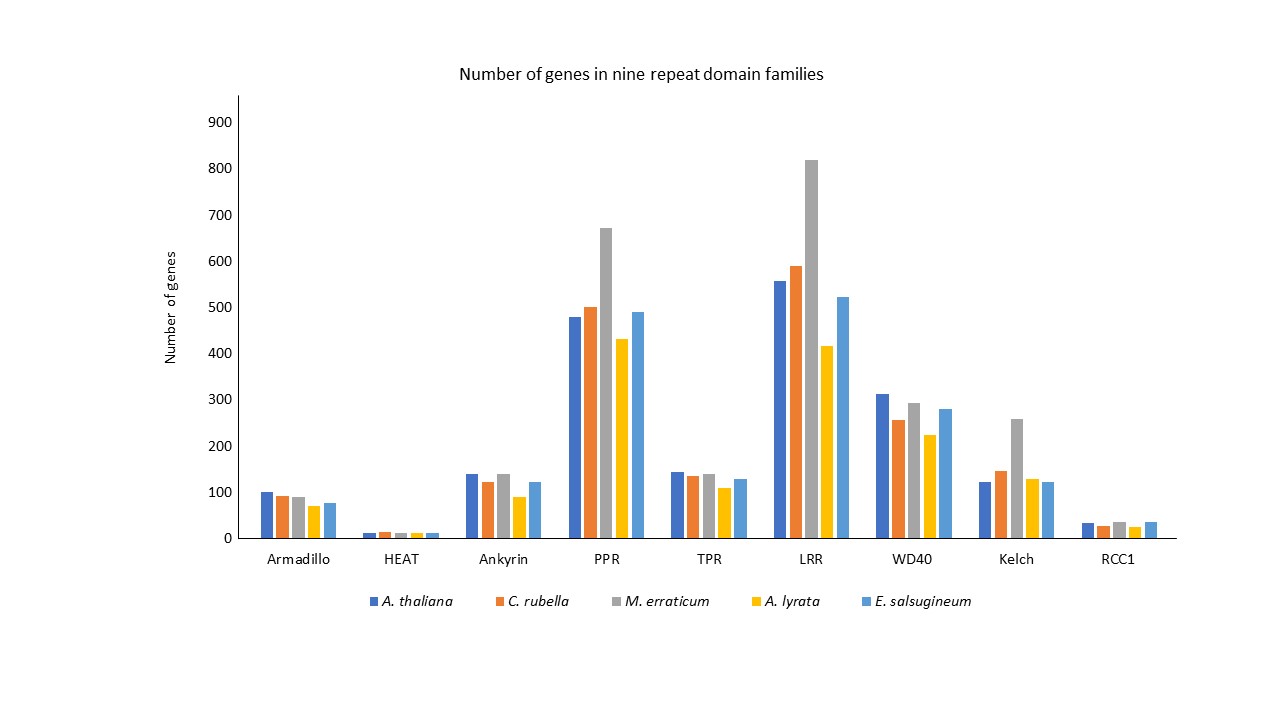

Supplement: Supplementary file 3 [file Image_2.jpeg]

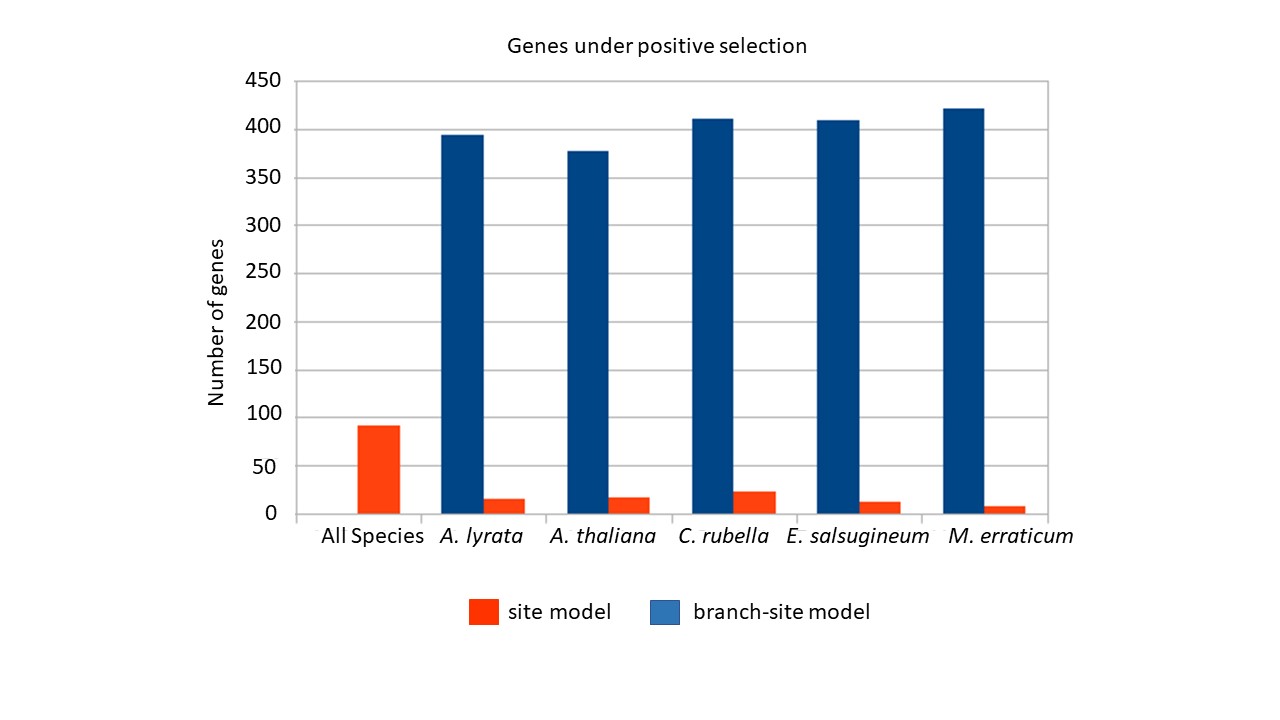

Supplement: Supplementary file 4 [file Image_3.jpeg]

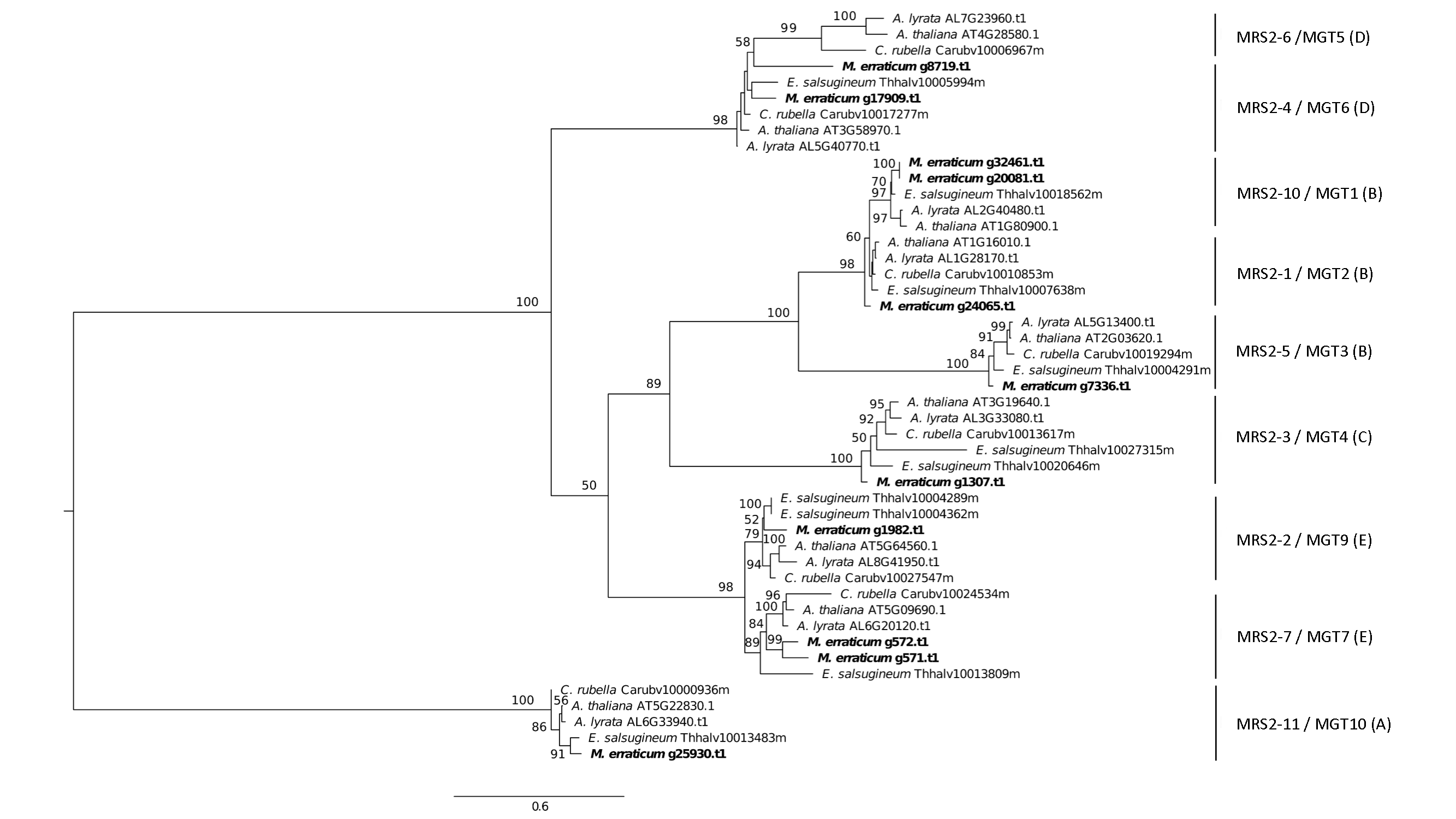

Supplement: Supplementary file 5 [file Image_4.tif]

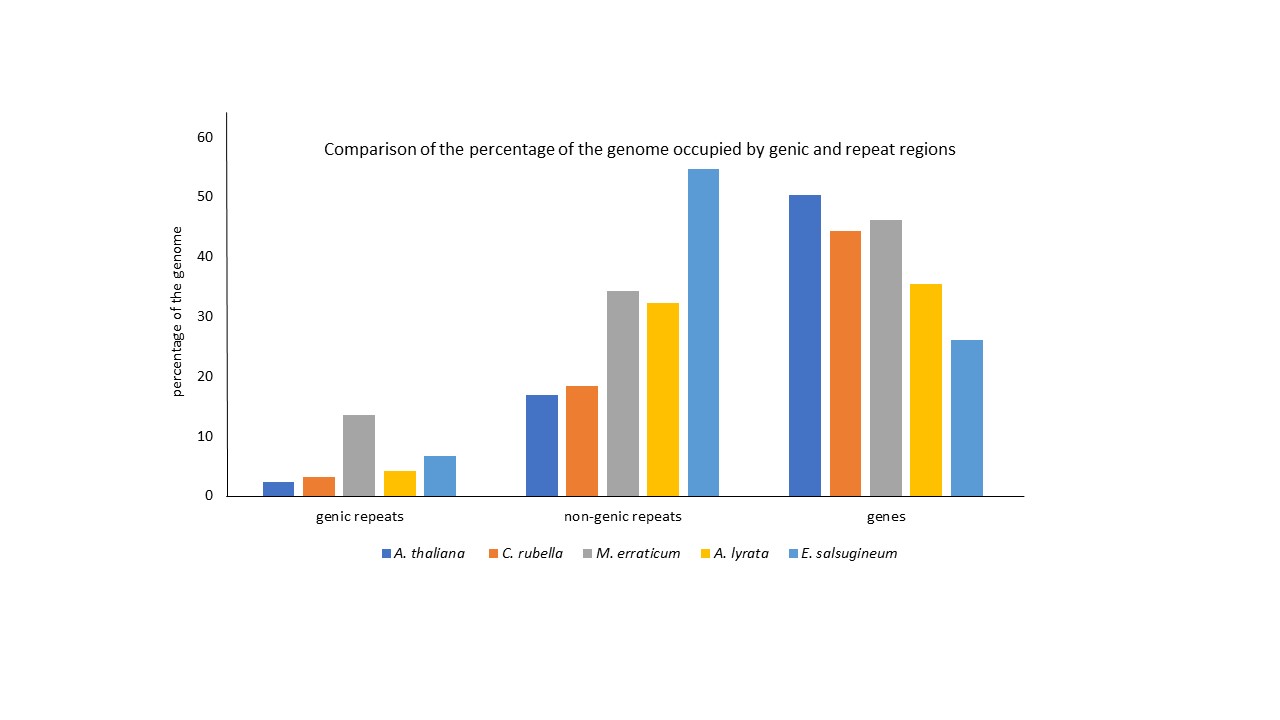

Supplement: Supplementary file 6 [file Image_5.jpeg]
